# Biomechanical Properties of Hemlocks: A Novel Approach to Evaluating Physical Barriers of the Plant–Insect Interface and Resistance to a Phloem-Feeding Herbivore

**DOI:** 10.3390/insects5020364

**Published:** 2014-06-03

**Authors:** Paul Ayayee, Fuqian Yang, Lynne K. Rieske

**Affiliations:** 1 Department of Entomology, University of Kentucky, S-225 Ag North, Lexington, KY 40546, USA; 2 Department of Chemical and Materials Engineering, University of Kentucky, Lexington, KY 40506, USA; E-Mail: fyang0@engr.uky.edu

**Keywords:** hemlock woolly adelgid, constitutive resistance, leaf cushion, microindentation

## Abstract

Micromechanical properties that help mediate herbivore access may be particularly important when considering herbivorous insects that feed with piercing-sucking stylets. We used microindentation to quantify the micromechanical properties of hemlock, *Tsuga* spp., to quantify the hardness of the feeding site of the invasive hemlock woolly adelgid, *Adelges tsugae*. We measured hardness of the hemlock leaf cushion, the stylet insertion point of the adelgid, across four seasons in a 1 y period for four hemlock species growing in a common garden, including eastern, western, mountain, and northern Japanese hemlocks. Leaf cushion hardness was highest in the fall and winter and lowest in summer for all species. Northern Japanese hemlock had relatively greater hardness than the remaining species. Our data contributes an additional perspective to the existing framework within which greater susceptibility and subsequent mortality of eastern hemlocks is observed. The potential application of microindentation to understanding the nature and relevance of plant mechanical defenses in plant–herbivore interactions is also demonstrated and highlighted.

## 1. Introduction

Plants employ a range of constitutive and induced defenses to deter insect herbivory. Constitutive defenses rely on inherent preexisting qualities to protect against herbivore attack, resulting in less damage relative to plants lacking these qualities [1]. In contrast, induced defenses are more specific and are activated via external stimuli, resulting in differential damage [2]. Plant resistance mechanisms may be chemical [3], nutritional [4], phenological [5,6,7], or mechanical [8]. Each mechanism may contribute to overall herbivore resistance, suggesting a link between resistance mechanisms and plant genetic composition, and several mechanisms may act in concert [6].

Seasonal and developmental variations in plant properties, such as foliar nutrients, defensive compounds, and toughness, correlate with seasonal herbivory [9,10,11,12]. For example, foliar toughness helps define the phenological window of opportunity for spruce budworm, *Choristoneura fumiferana* (Clemens) (Lepidoptera: Tortricidae) feeding on white spruce, *Picea glauca* (Moench) Voss, demonstrating a link between host plant phenology, needle toughness, and herbivore susceptibility [6]. Foliar toughness varies within and between taxa, among age classes, and with environmental variability [8].

Plant micromechanical properties have been cited as evidence of antiherbivore defense [8,13,14], but the basis for this mode of resistance is not well studied. This lack of attention appears to be due to difficulties inherent in characterizing plant micromechanical properties. This is due in part to the heterogeneous, composite and anisotropic nature of plant tissues [14], misapplication of the terms ‘toughness’ and ‘hardness’ and their measurements [13], and to inadequate instrumentation used to measure these properties. 

The application of force on a material produces a displacement in the direction of the force, which has consequences for the shape and integrity of the material [13]. Hardness is defined as force per unit area (MPa) required to cause permanent material deformation due to the contact from a sharp object. Toughness refers to a material’s ability to resist deformations and is defined as the energy consumed in generating a deformation (KJm^−2^), *i.e.*, force applied multiplied by the displacement of the material [13].

Plant micromechanical properties (*i.e.*, toughness) in ecological studies have historically been measured using penetrometers, which measure the force needed to puncture specific plant tissue [15,16,17,18,19]. Penetrometers rely on application of a weighted material on top of a sharp or blunt object such as a pin. The weight required to puncture the test material is taken as the toughness of the material, and is usually represented as newtons (N), grams (g), or in some cases gcm^2^, instead of the appropriate unit, KJm^−2^. Obvious issues with penetrometer readings include lack of a standardized process for quantifying target measurements, lack of detail in construction, superficial use of the measures generated, and the realization that what is measured is not technically ‘toughness’ [13,20,21]. Alternatives such as razor slicing have also been used to estimate leaf fracture toughness [21], but both approaches have drawbacks [14,21] that limit their ability to reliably measure the biomechanical properties relevant in insect-plant relations.

Microindentation, a depth sensing procedure used to characterize mechanical properties of materials on a fine scale and with a high degree of accuracy [22], may provide a novel approach to evaluating the interface between insect herbivores and their host plants, and to explore with greater accuracy and resolution the mechanical dimensions underlying host plant resistance mechanisms. Microindentation employs diamond pyramidal or spherical tip indenters to produce local deformations on a specified material, and then measures the resistive force of the material to the deformation as a function of the depth or area of the deformation [23]. It measures specific physical attributes of a given material with much greater precision than traditional penetrometers. Additionally, other micromechanical properties such as the elastic modulus, energy of indentation, and stiffness of materials can be determined from the load-displacement curve generated from the indentation process. Microindentation has the added advantage of being an automated and standard process that facilitates easy replication and repeatability; it is relatively fast [24] and non-destructive [22].

Microindentation approaches have previously been used to assess plant tissue characteristics relevant to wood processing and plant structure studies [25,26,27,28,29]. Its application for evaluating micromechanical properties of the penetration point for stylet-inserting herbivores such as aphids, stink bugs and the hemlock woolly adelgid, however, has not been investigated.

**Figure 1:**
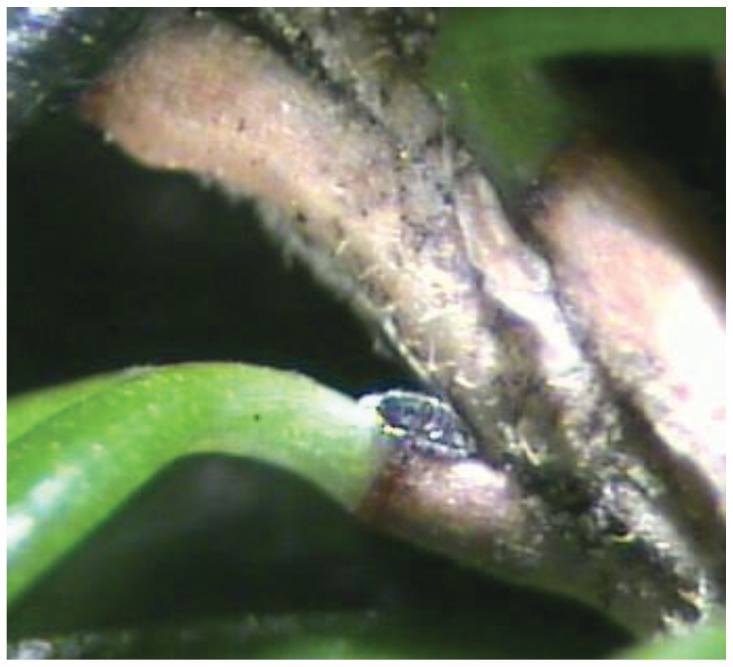
Hemlock woolly adelgid nymphs insert their feeding sylet proximal to the abscission layer at the base of the hemlock leaf (leaf cushion). Photo: Lori A. Nelson.

The hemlock woolly adelgid (HWA), *Adelges tsugae* (Annand) (Hemiptera: Adelgidae), is an invasive forest pest in eastern North America. Hemlocks vary in their suitability for and susceptibility to adelgid colonization and feeding [30,31,32,33,34], but the adelgid causes extensive mortality of the highly susceptible eastern, *Tsuga canadensis* (L.) Carrière, and Carolina, *T. caroliniana* Engelm, hemlocks. Adults are sessile and are recognized by the presence of a white woolly ovisac. Newly hatched nymphs (crawlers) of both generations actively disperse to suitable feeding sites. The adelgid feeding stylet is about three times the length of the adult adelgid [35], and is inserted into the plant immediately proximal to the abscission layer at the base of the hemlock leaf (leaf cushion) (Figure 1). The stylet penetrates deep through the vascular tissues of the leaf cushion, accessing starches stored in the xylem ray parenchyma cells at the leaf base [35]. Feeding depletes starch reserves, leading to needle loss, twig and branch dieback, and causing tree mortality [34,36].

Because adults are sessile, the ability of the crawlers to locate and penetrate suitable leaf cushions is critical to colonization success. Ontogenetic or phenological differences in the feeding site during periods of crawler activity may provide a basis for hemlock woolly adelgid resistance among hemlocks [30,31,33,37]. In this study we investigated the application of microindentation to elucidate the role of biomechanical properties toward resistance in hemlocks to adelgid herbivory, and also quantified species-specific and seasonal differences in hardness of the adelgid feeding site for four hemlock species over a one-year period. Ultimately we sought to determine whether the susceptibility of eastern hemlock to the hemlock woolly adelgid might be influenced by micromechanical properties of the adelgid stylet insertion point.

## 2. Experimental Section

### 2.1. Experimental Design

Four hemlock species were planted in winter 2007 in a common garden at the University of Kentucky Spindletop Research Farm, including eastern hemlock, western hemlock, *T. heterophylla* (Rafin.) Sarg., mountain hemlock, *T. mertensiana* (Bong.) Carrière, and northern Japanese hemlock *T. diversifolia* (Maxim.) Mast. Trees were planted as 3–4 year old containerized nursery stock in a common garden at 3 m intervals in a randomized block design. Each block (n = 10) contained a single tree of each species arranged randomly. Trees were fertilized with 15–15–15, N–P–K slow release fertilizer with a 3 month release duration (Osmocote, Sierra Chemical, Milipitas, CA, USA) at planting. Competing vegetation was suppressed with landscape fabric (DuPont, Wilmington, DE, USA). Trees were protected with 70% shade cloth (Dewitt, Sikeston, MO, USA) and watered at 2–3 week intervals throughout the summer months. 

### 2.2. Sampling

To evaluate species-specific and seasonal changes in micromechanical properties of the leaf cushions, three blocks containing all four hemlock species were randomly selected. Branch tips were removed from the apical region in the upper third of the south side of each tree, and separated into current- *versus* previous-year (new *versus* old) growth, generating three current-growth twigs and three previous-growth twigs per tree. Needles were removed using a scalpel, taking care to excise them distal to the abscission layer and leaving the leaf cushions intact. The twigs, with leaf cushions intact, were then stabilized on a glass microscope slide by immobilizing them in a 4.5 mL aliquot of a synthetic polymer composed of a 2:1 ratio of Sample Kwik Powder: Sample Kwik liquid (Buehler Ltd., Lake Bluff, IL, USA), so that the leaf cushions themselves were exposed. A single species (N = 3 trees) was sampled on each day, so that microindentations were performed on all four species over four consecutive days. 

Sampling and subsequent microindentations for each replicate of each species (N = 3 trees) were completed in a single day, and repeated four times during each season. Sampling was conducted in spring (24 March–10 April), as progrediens eggs and crawlers were generated, summer (7–26 July), during sistens’ aestivation, fall (18–23 November), coinciding with emergence of sistens nymphs from aestivation, and winter (9–13 February), during sistens’ development. 

### 2.3. Microindentations

**Figure 2:**
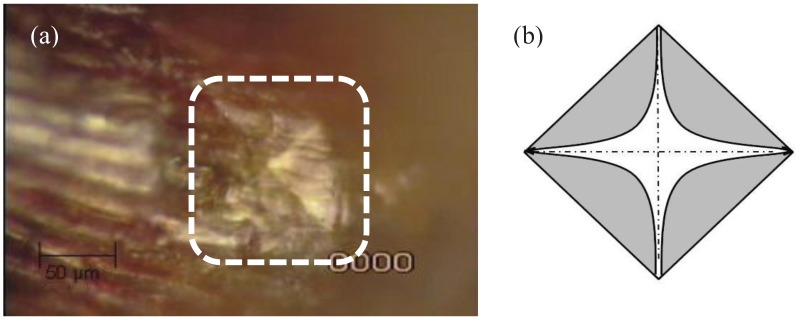
(**a**) Actual and (**b**) diagrammatic indentation mark resulting from the microindentation process of a CSM pyramidal micro-indenter.

Micromechanical properties of leaf cushions were measured using a micro-indenter (CSM Instruments, Needham, MA, USA) in the Micromechanics Laboratory at the University of Kentucky. Indentation tests were performed using a Vickers diamond indenter, creating an indentation on the leaf cushions following application of a load (Figure 2). 

**Figure 3:**
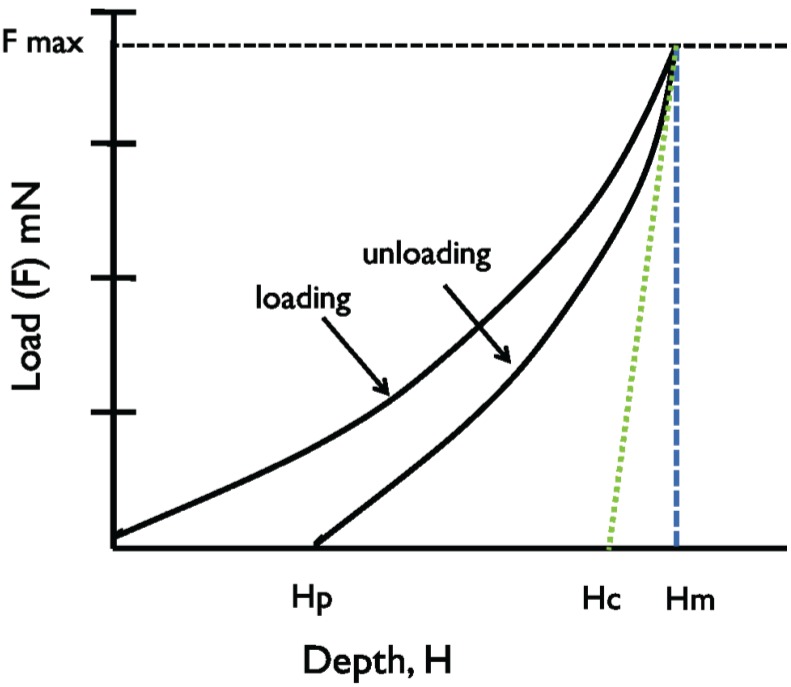
A schematic representation of a typical indentation process, demonstrating the relationship between indentation depth and load, where Hp: final depth after load removal, Hr: residual depth after load removal, Hc: contact depth of indentation, and Hm: maximum depth of indentation.

Following the indentation, hardness is then determined using the load used to create the indentation and the calculated area of the impression on the material (Figure 3) [22].

Indentations were performed directly on the leaf cushions (Figure 2a) attached to the twigs that were embedded in the polymer on the glass microscope slides. Two loads were used (30 and 45 mN), with constant loading and unloading rates of 120 N/min with no holding time/ pause for the loading phase and the unloading phase. There were two replications per load. For each species, there were twelve 30 mN and 45 mN indentations, making a total of 24 indentations per species. Indentations were repeated 4 times for each species during each season for a total of 96 indentations per species per season. Thus, there were 384 indentations for each species over four seasons.

Because the fluid-like behavior of plant material resists deformation, a confounding factor in this approach is the inability to consistently obtain indentation marks on leaf cushions. The indentation mark is needed for calculation of the area of deformation following indentation at a particular load, in order to determine hardness (Figure 3). Knowing the actual area of an impression is essential to directly obtain the true mechanical properties of the material being evaluated [22]; it is calculated by determining the square of the average diagonal lengths of the indentation mark. Consequently we used an alternate approach to determine the projected area of impression/ indentation using contact depth (Hc) (Figure 3), a parameter in the indentation process. The projected contact area (mm^2^) for the pyramidal indenter we used is calculated as

A = 24.5 (Hc)²

where Hc = contact depth of indentation (mm), or the vertical distance from the edge of the contact area to the indenter tip [22].

Hardness is determined following indentation using the load of indentation and the calculated area of the impression on the material [22] (Figure 2). Leaf cushion hardness (N/mm^2^) or (MPa) is then calculated using

H = P_max_∙A^−1^;

where H = hardness, A = projected contact area (mm²), and P_max_ = Load (N).

Microindentation measurements can also be confounded by an indentation size effect, or a decrease in hardness with increasing applied load [22], which can be caused by elastic recovery of the material, potential for a mixed elastic/plastic deformation response of the test material, and indenter-specimen friction resistance coupled with elastic resistance of material [38]. Any indentation size effect in our samples would likely be attributable to the heterogeneous and anisotropic nature of our target material, coupled with its high viscoelastic feature, relative to the harder, homogeneous, and more rigid materials used in conventional microindentation [22,38]. Additional concerns for our indentation process might arise from the unevenness of our leaf cushion surfaces, the initial depth of penetration, compliance of the system, and noise associated with unloading [39].

For any hardness determination there are ‘load-dependent’ and ‘load-independent’ components; we utilized a load-dependent approach for our determinations. This approach uses average hardness values determined using multiple test loads to account for any decrease in hardness with increasing load of indentation. In this approach, the Y-axis values (Figure 3) are the ‘load-dependent hardness’, *i.e.*, the hardness determined at particular loads, and reflects actual hardness of the material being tested. A second approach is to apply multiple loads on the same material and subsequently regress the determined hardness values against the tested indentation loads. The negative slopes of the resulting lines are an indication of the rate of change of the hardness with increasing loads, and the intercepts are the hardness estimates as the indentation load approaches zero, or the ‘load-independent hardness’. The nature of our plant material precluded the use of load-independent hardness, so in this study we focused on load-dependent hardness, and values obtained at loads of 30 mN and 45 mN were averaged for the four hemlock species across four seasons. 

### 2.4. Statistical Analysis

Trees were our unit of replication, generating a total of 96 data points (3 trees × 4 species × 4 sample intervals × 2 growth classes). Hardness values from microindentations were calculated using the projected area approach, and data for both loads and hardness values were log transformed for subsequent analysis. A repeated measure ANOVA was performed using species, load, tree and tissue age as model effects, with season as the repeated measure. This approach allowed us to evaluate effects over time with the absence of independence between measurements on the same treatment unit (tree). Indentation load, tree and tissue age accounted for the majority of the variability in the data; treating them as fixed effects enabled the species effect to be determined with greater precision. Means comparisons were then carried out for significant effects using the standard least squares model with seasonal hardness as the dependent variable and species, age, and load as model factors. Means separations were carried out using Tukey’s HSD. All analyses were performed using JMP^®^ 10.0.

## 3. Results and Discussion

Microindentations at loads of 30 and 45 mN yielded hemlock leaf cushion hardness values with comparable variability for all species, supporting the feasibility of utilizing this approach to evaluate relevant plant micromechanical properties, appropriately optimized for target test materials. 

Our overall model evaluating leaf cushion hardness was significant (F = 47.23; df = 7, 40; *p* < 0.0001), including the effects of hemlock species (F = 14.06; df = 3, 40; *p* < 0.0001), tissue age (F = 6.09; df = 1, 40; *p* = 0.018), tree (F = 7.29; df = 2, 40; *p* = 0.0020), and indentation load (F = 267.7; df = 1, 40; *p* < 0.0001), in the repeated measures analysis. 

Not surprisingly, all four hemlock species demonstrated significant seasonal changes in leaf cushion hardness over the course of sampling (F = 9.82; df = 3, 38; *p* < 0.0001). Winter and spring leaf cushion hardness values were comparable, followed by a decrease in summer, and subsequent increase to an autumn maximum (Table 1). 

**Table 1:** Seasonal hardness (mean ± s.e.) (MPa) of the hemlock woolly adelgid feeding stylet insertion point across four *Tsuga* species. Means separation performed on log-transformed data. Means followed by the same letter do not differ (*p* < 0.05).

Season	Hardness (MPa)
Spring	83.17 ± 0.02 b
Summer	47.71 ± 0.03 c
Autumn	158.48 ± 0.03 a
Winter	95.50 ± 0.02 b
F; df = 3, 38/*p*	9.82/<0.0001

We found significant species-specific variation in leaf cushion hardness between seasons (F = 14.06; df = 3, 40; *p* < 0.0001), with northern Japanese hemlock having significantly greater hardness values across seasons (Table 2). 

**Table 2:** Species-specific hardness (mean ± s.e.) (MPa) of four *Tsuga* species at the feeding stylet insertion point of the hemlock woolly adelgid, *Adelges tsugae*. Means separation performed on log-transformed data; means followed by the same letter do not differ (*p* < 0.05).

Hemlock species	Hardness (MPa)
Eastern, *T. canadensis*	87.09 ± 0.04 ab
Northern Japanese, *T. diversifolia*	104.71 ± 0.04 a
Mountain, *T. mertensiana*	81.28 ± 0.04 b
Western, *T. heterophylla*	77.62 ± 0.03 b
F; df = 3, 40/*p*	14.60/<0.0001

There was a significant season × species interaction (Wilks’ Lambda F = 3.82; df = 9, 92; *p* = 0.0004), driven by differences in autumn and winter hardness (Table 3). 

**Table 3:** Hardness (mean ± s.e.) of the hemlock woolly adelgid feeding stylet insertion point of four *Tsuga* species across four seasons. Means separation performed on log‑transformed data; means within columns followed by the same letter do not differ (*p* < 0.05).

Hemlock species	Hardness (MPa)			
	Spring	Summer	Autumn	Winter
Eastern, *T. canadensis*	89.12 ± 0.05 a	46.77 ± 0.05 a	151.35 ± 0.06 bc	93.32 ± 0.04 b
N. Japanese, *T. diversifolia*	74.13 ± 0.05 a	51.28 ± 0.06 a	213.80 ± 0.05 a	147.91 ± 0.05 a
Mountain, *T. mertensiana*	81.28 ± 0.05 a	43.65 ± 0.05 a	165.95 ± 0.05 ab	77.62 ± 0.04 b
Western, *T. heterophylla*	85.11 ± 0.04 a	43.65 ± 0.05 a	123.03 ± 0.05 c	83.18 ± 0.03 b
F; df = 3, 44/*p*	0.89/0.45	0.77/0.52	11.35/0.0001	13.68/0.0001

Northern Japanese hemlock leaf cushion hardness was highest and western hemlock leaf cushion hardness was lowest in the autumn. In the winter, however, northern Japanese hemlock was significantly higher than all the other hemlocks evaluated (Table 3). No significant differences among species were evident in spring and summer, when hardness values were relatively low. 

There were significant season × age (F = 3.87; df = 3, 38; *p* < 0.0001) and season × tree (Wilks’ Lambda F = 3.42; df = 6, 76; *p* < 0.0001) interactions in the repeated measures analysis. These most likely reflect differential maturation of twigs of different species across seasons, and between different trees of a species, reflected by higher hardness values in the fall and winter compared to spring and summer.

Lastly, there was a significant effect of microindentation load (F = 267.7; df = 1, 40; *p* < 0.0001) that suggest an indentation size effect; this was addressed in our study by using average hardness estimates across both loads in determining the species and seasonal effects and interactions, rather than a single hardness reading at a specific load. 

## 4. Discussion and Conclusions

Our data demonstrate that microindentation can provide a repeatable and reliable means of evaluating micromechanical behavior of a heterogeneous, composite biological material [14]. Using the predicted area approach to overcome the presence of an indentation size effect [22], we generated consistent and repeatable results for our test materials. We demonstrate temporal changes in hemlock leaf cushion hardness, but these differences differ from the seasonal increases in tissue hardness evident in some systems [5,9,11,12,17]. Leaf cushion hardness across hemlock species was lowest in the summer and greatest in the autumn, perhaps reflecting tissue maturation following spring growth and summer dormancy [40]; leaf cushion hardness was intermediate in the winter and spring. 

Hemlock woolly adelgid crawler survival may be compromised due to difficulties inherent in penetrating leaf cushions. Greater difficulty in stylet penetration, whether because the material is too hard or too soft, may hinder adelgid colonization on resistant hemlocks, and/or minimize the negative effects of adelgid feeding on the hemlock host. The intermediate hardness in early spring (late March to early April) coincides with spring generation adelgid crawler activity, and occurs before hemlock budbreak and leaf expansion. Thus, all tissues sampled during our spring sample interval were from the previous year’s growth [41], which may explain the absence of differences in hardness based on tissue age. Although our focus in this study was on the hemlock host plant and no bioassays were conducted, adelgid crawlers of the spring generation preferentially settle on the same age tissue on which their parents fed [42]; our findings corroborate this. Oten *et al.* [33,37] evaluated six *Tsuga* species and a hybrid, and found that the hemlock leaf cuticle was thinnest near the point of adelgid stylet insertion relative to other locations measured, and this might relate to adelgid host selection and/or hemlock susceptibility. It follows that cuticle thickness could relate to leaf cushion hardness.

Physical characteristics of the plant–insect interface are known to play a role in other systems. The aphids *Myzus persicae* (Sulz.) and *Nasonovia ribisnigri* (Mosley) (Hemiptera: Aphididae), make a greater number of shorter probes on resistant lettuce, *Lactuca* *sativa* (Fam: Asteraceae), than on susceptible plants, and shorter probes lead to less successful feeding [42]. Leaf toughness of the herbaceous *Aristolochia kaempferi* (Fam: Aristolochiaceae) prolonged larval development, decreased larval survival, and lengthened pupal diapause in *Byasa alcinous* (Fam: Papilionidae), a mandibulate folivore [12]. Similar findings were reported for the endophagous *Bagous hydrillae* (Fam: Curculionidae) on aquatic *Hydrilla* sp. [11]. Studies have demonstrated a link between host phenology, foliar toughness, and herbivore susceptibility. Foliar toughness is correlated with phenological development, and has been shown to play a role in herbivore susceptibility of neotropical shrubs [43], temperate evergreen trees [44], and temperate deciduous trees [17,45].

Plant chemistry likely also influences hemlock susceptibility to hemlock woolly adelgid [41,46,47]. Lagalante and Montgomery [41] show that eastern and Carolina hemlocks have fluctuating levels of specific terpenes that may be linked to adelgid life history and influence adelgid behavior. It is conceivable that phytochemical defenses may be mediating resistance to adelgid during certain times of the year, and as hemlocks mature, micromechanical properties of the feeding site become increasingly responsible for mediating resistance.

We demonstrate the feasibility of utilizing a novel approach to evaluate plant micromechanical properties that may contribute to constitutive defenses against phloem-feeding herbivores. Here we focus on eastern hemlock and the invasive hemlock woolly adelgid, but our approach may be applicable to other plant–herbivore systems. Given the role of eastern hemlock as a foundation species in eastern North American forests, a greater understanding of the mechanisms of plant resistance, in concert with biological control efforts, may enhance our ability to mitigate the devastating effects of this invasive herbivore. 
